# Rutaecarpine Promotes Adipose Thermogenesis and Protects against HFD-Induced Obesity via AMPK/PGC-1α Pathway

**DOI:** 10.3390/ph15040469

**Published:** 2022-04-13

**Authors:** Dandan Chen, Yanan Duan, Shuxiang Yu, Xinwen Zhang, Ni Li, Jingya Li

**Affiliations:** 1 School of Life Sciences, Shanghai University, Shanghai 200444, China; dandan_chen@shu.edu.cn (D.C.); shuxiang_yu@shu.edu.cn (S.Y.); 2 State Key Laboratory of Drug Research, The National Center for Drug Screening, Shanghai Institute of Materia Medica, Chinese Academy of Sciences, Shanghai 201203, China; duanyanan@simm.ac.cn (Y.D.); xwzhang@simm.ac.cn (X.Z.); lini@simm.ac.cn (N.L.)

**Keywords:** obesity, rutaecarpine, adipocytes, thermogenesis, peroxisome-proliferator-activated receptor γ co-activator-1α, AMP-activated protein kinase, browning

## Abstract

Pharmacological activation of adaptive thermogenesis to increase energy expenditure is considered to be a novel strategy for obesity. Peroxisome-proliferator-activated receptor γ co-activator-1α (PGC-1α), which serves as an inducible co-activator in energy expenditure, is highly expressed in brown adipose tissues (BAT). In this study, we found a PGC-1α transcriptional activator, natural compound rutaecarpine (Rut), which promoted brown adipocytes mitochondrial biogenesis and thermogenesis in vitro. Chronic Rut treatment reduced the body weight gain and mitigated insulin sensitivity through brown and beige adipocyte thermogenesis. Mechanistic study showed that Rut activated the energy metabolic pathway AMP-activated protein kinase (AMPK)/PGC-1α axis, and deficiency of AMPK abolished the beneficial metabolic phenotype of the Rut treatment in vitro and in vivo. In summary, a PGC-1α transcriptional activator Rut was found to activate brown and beige adipose thermogenesis to resist diet-induced obesity through AMPK pathway. Our findings serve as a further understanding of the natural compound in adipose tissue and provides a possible strategy to combat obesity and related metabolic disorders.

## 1. Introduction

Obesity has reached epidemic proportions globally, with at least 2.8 million people dying each year due to overweight or obesity [1,2]. Adipose tissue serves as an energy terminal, a warehouse where excess nutrients are stored in the form of triglycerides (TG), which stores and releases lipids under the influence of external or internal cues [3]. Mitochondria are essential for adipose tissue functions, which burn fuels to produce energy in the form of ATP through oxidative phosphorylation (OXPHOS) or heat through uncoupling proteins to regulate lipid turnover and maintain metabolic homeostasis [4]. The regulations of mitochondria activity in the process of white adipose tissue (WAT) remodeling and BAT thermogenesis are increasingly appreciated. Many studies have shown that activating brown and white adipocytes thermogenesis could be a possible practical strategy to treat obesity and associated comorbidities.

PGC-1α, an inducible transcriptional co-activator, is highly expressed in metabolically active tissues, including skeletal muscle, heart, kidney and BAT [5,6]. Except for the major role in stimulating mitochondrial biogenesis, PGC-1α promotes thermogenic program in brown and beige adipocyte via interacting with peroxisome proliferator-activated receptor γ (PPARγ) [7], retinoic acid receptor (RAR) [8] and thyroid receptor (TR) [9] to enhance *Ucp1* transcription. Regulation of PGC1s primarily relies on post-translational modifications (PTMs) and subsequent regulation of positive feedback transcriptional loops. After several decades of research, many modification enzymes and small molecules have been reported to control brown and white adipose function through PGC-1α; these pathways include p38 MAPK [10,11], AMPK [12,13], Sirt1 [14,15], PRMT1 [16], JMJD3A [17], etc. and external cues include AMPK agonists [18,19,20], MAPK agonists [21] and Sirt1 agonists [22,23].

Natural molecules provide structural inspiration for drug development. Through promoter luciferase reporter assay, we obtained a natural compound, rutaecarpine (Rut), which enhanced *Pgc-1α* transcription obviously. Rut is a bioactive alkaloid isolated from herb *Evodia rutaecarpa* (Wu Zhu Yu) [24,25] and its chemical structure is 8,13-dihydroindolo-[2′,3′:3,4]pyrido[2,1-b]quinazolin-5(7H)-one. The dried unripe fruit of *Evodia rutaecarpa* has been recorded in multiple versions of Chinese Pharmacopoeia and it could be used either alone or in combination with other herbal medicines to cure headache, epigastric pain, menorrhalgia, dermatophytosis, celialgia, emesis and aphtha [26]. As one of the most representative indolopyridoquinazoline alkaloids of *Evodia rutaecarpa*, Rut has also been found to possess many kinds of biological functions, such as anticancer, anti-inflammatory [27] and analgesic, as well as effects on the cardiovascular [28,29] and endocrine systems. Biochemical and pharmacological studies have illustrated the molecular targets of rutaecarpine, such as TRPV1, CGRP, AMPK, ABCA1 and β1-AR [25]. Here, Rut displayed a significant activity in promoting thermogenesis and increasing oxygen consumption in both brown and beige adipocytes. In vivo, Rut treatment reduced body weight gain and mitigated insulin sensitivity in high-fat diet (HFD)-induced obese mice. Further investigation revealed that activation of AMPK/PGC-1α pathway contributed to the improved metabolic phenotypes by Rut.

## 2. Results

### 2.1. Rut Activates Expression of PGC-1α and Induces Thermogenic Program in C3H10-T1/2

**Figure 1:**
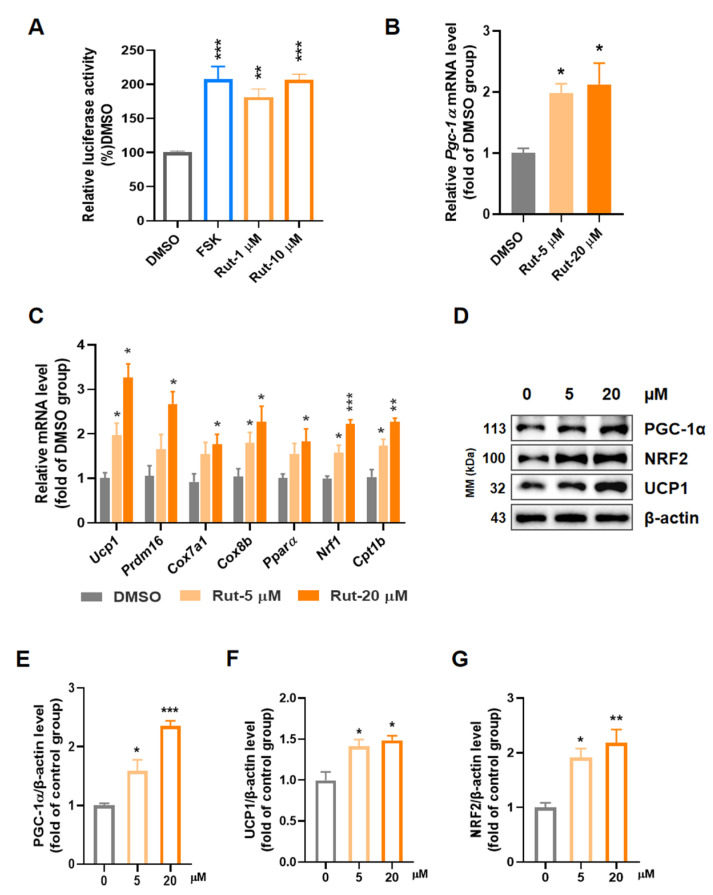
Rut increases expression of PGC-1α and drives the thermogenesis program. (**A**) The screening result of Rut in activating PGC-1α. (**B**) Effect of Rut on activating PGC-1α in C3H10-T1/2. (**C**) RT-qPCR analysis of genes indicated in C3H10-T1/2. (**D**) Expressions of protein indicated in C3H10-T1/2 treated by Rut for 24 h. (**E**–**G**) Relative protein level of PGC-1α (**E**), UCP1 (**F**) and NRF2 (**G**) compared to β-actin. *n* = 3 per group. Data are presented as the means ±SEM. * *p* < 0.05, ** *p* < 0.01, *** *p* < 0.001, Rut groups versus vehicle group by one-way ANOVA.

PGC-1α serves as a transcriptional co-activator in mitochondrial biogenesis and thermogenesis in brown and beige adipocytes and then curbs obesity. By natural products screening via PGC-1α reporter cell line reported before [30], we obtained a small compound Rut, which increased luciferase activity at 1.8- and 2.1-fold for 1 and 10 μM (Figure 1A) without obvious cytotoxicity (Figure S1A). Forskolin (FSK), which was reported to stimulate expression of PGC-1α [30,31], increased PGC-1α promoter reporter by 2.1-fold on 10 μM. We then investigated the thermogenic effect of Rut in classical brown adipocytes C3H10-T1/2. After treatment with Rut, the mRNA expression of *Pgc-1α* was obviously elevated (Figure 1B), and thermogenic genes, such as peroxisome proliferator-activated receptor α (*PPARα*), cytochrome c oxidase Subunit 7a1 (*Cox7a1*) and cytochrome c oxidase subunit 8b (*Cox8b*), were also increased (Figure 1C). Collectively, the protein level of UCP1 was substantially increased after Rut treatment, together with thermogenic transcriptional factor PGC-1α and nuclear factor E2-related factor 2 (NRF2) (Figure 1D–G). Moreover, the mitochondrial DNA copy number also increased (Figure S1B), indicating that Rut could enhance mitochondrial biogenesis. Taken together, these results suggest that Rut triggers the expression of PGC-1α and promotes thermogenesis in C3H10-T1/2.

### 2.2. Rut Enhances Brown and Beige Adipocytes Thermogenesis Program and Mitochondrial Respiration

**Figure 2:**
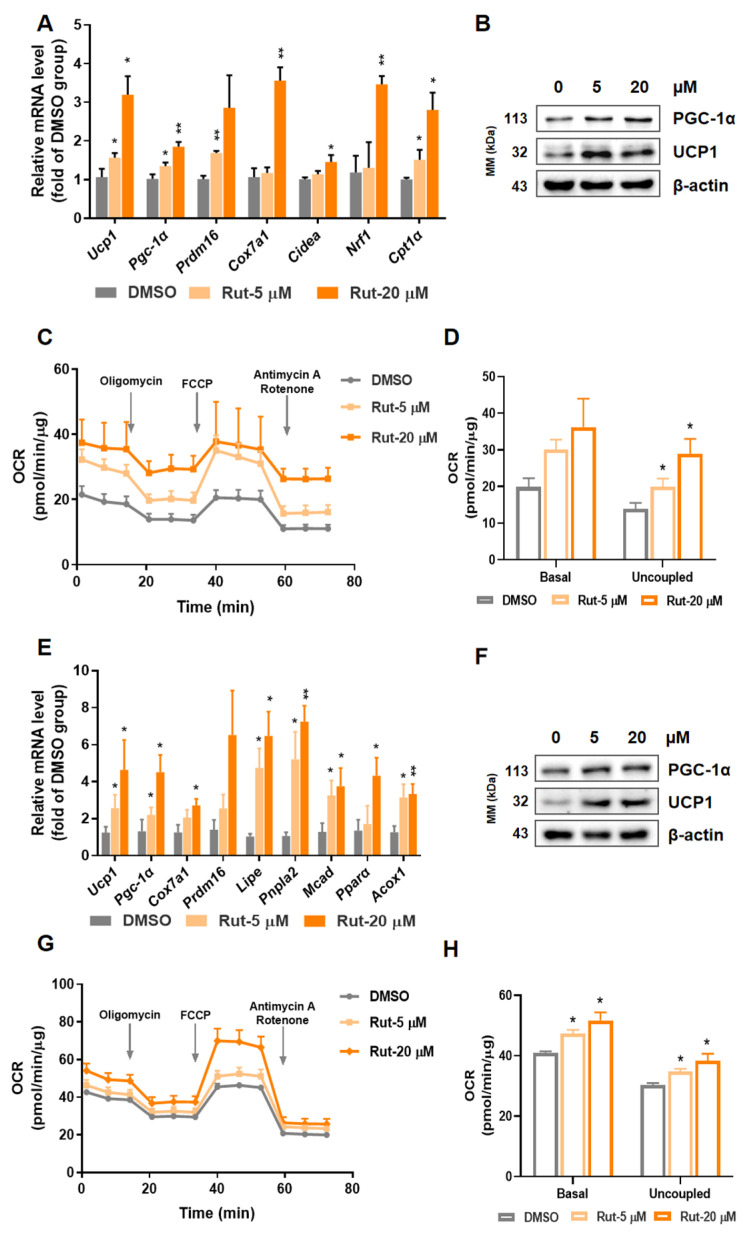
Rut enhances thermogenic program and increases mitochondrial respiration of primary brown and beige adipocytes. (**A**) Expression of thermogenic genes in differentiated primary brown adipocytes (BAT-SVF). (**B**) UCP1 and PGC-1α protein expression level in BAT-SVF. (**C**) OCR in differentiated primary brown adipocytes on basal condition, in the presence of 2 μM oligomycin, 1 μM FCCP or 1 μM rotenone/antimycin. (**D**) Statistical graphs of basal and uncoupled respiration in BAT-SVF. (**E**) Expression of thermogenic and fuel consuming genes in functional primary subcutaneous adipocytes (Sub-SVF). (**F**) Western blot analysis of PGC-1α, UCP1 protein levels in Sub-SVF. (**G**) The testing of OCR in Rut-treated Sub-SVF. (**H**) Statistical values of basal and uncoupled respiration in Sub-SVF. *n* = 3 per group. Data are presented as the means ±SEM. * *p* < 0.05, ** *p* < 0.01, Rut groups versus vehicle group by one-way ANOVA.

To verify the thermogenic property of Rut, stromal vascular fraction (SVF) from interscapular brown adipose tissue (iBAT) or inguinal white adipose tissue (iWAT) were isolated and induced for further exploration. In mature primary brown and beige adipocytes, the thermogenic relative factors were increased, including *Ucp1*, *Cox7a1*, *Pgc-1α*, PR domain containing 16 (*Prdm16*) and cell death-inducing DFFA-like effector a (*Cidea*) on the transcriptional level (Figure 2A), as well as UCP1 and PGC-1α on protein level (Figure 2B). Then, we tested cellular oxygen consumption rate (OCR) by using seahorse system. Compared to blank group, cells exposed to Rut showed a higher respiration ratio, in both basal and uncoupled state (Figure 2C,D). All above results demonstrate that Rut elevates thermogenesis program and mitochondrial respiration capacity in both brown and beige primary adipocytes.

### 2.3. Rut Protects against HFD-Induced Obesity and Improves Insulin Resistance 

**Figure 3:**
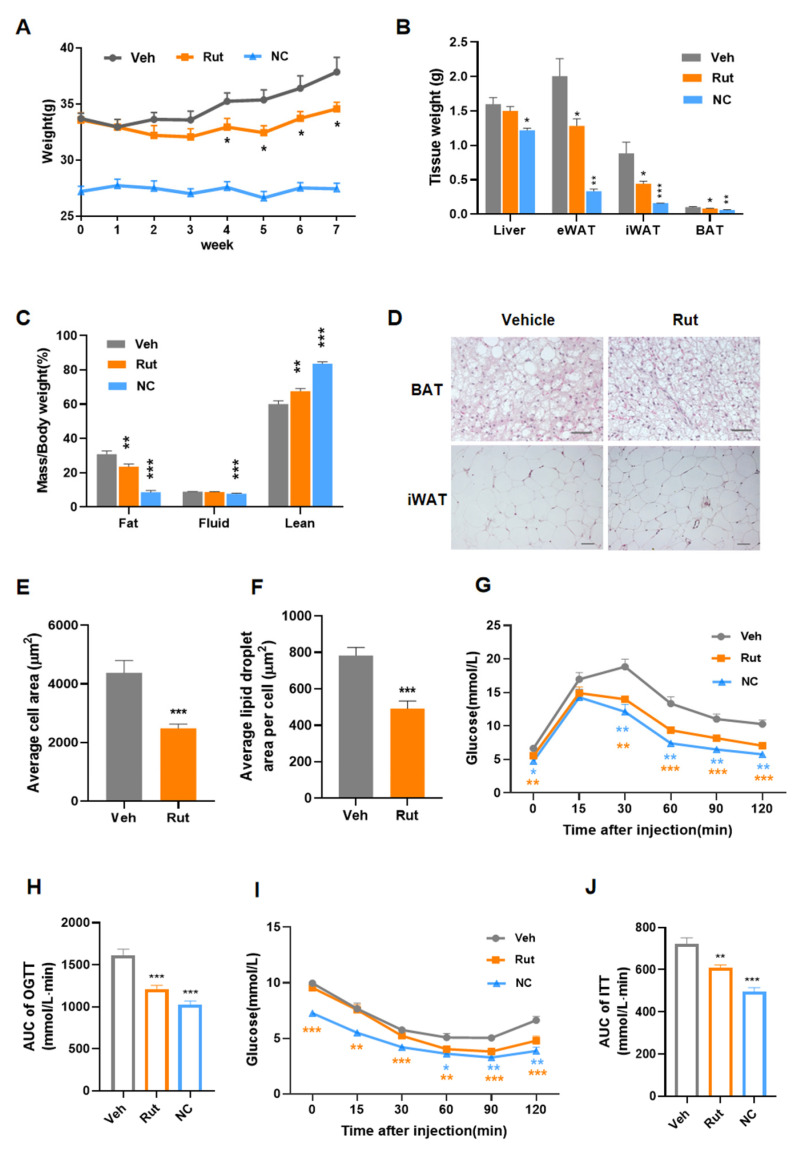
Rut restrains the weight gain of HFD-fed mice and ameliorates insulin resistance. (**A**) Body weights were recorded each week after Rut treatment. (**B**) Tissues were collected and weighted. (**C**) Proportion of body composition of mice. (**D**) Representative hematoxylin and eosin staining (HE staining) from interscapular BAT and inguinal WAT sections of HFD-fed mice after 8 weeks of Rut treatment. Scale bar, 50 µm. (**E**,**F**) Quantification of adipocyte area of iBAT (**E**) and iWAT (**F**). (**G**) Blood glucose curves during testing of oral glucose tolerance (GTT). (**H**) Area under curve (AUC) of GTT. (**I**) Blood glucose levels were plotted in insulin tolerance test (ITT). (**J**) Analysis of AUC during ITT. *n* = 6 per group. Data are presented as the means ±SEM. Student’s *t*-test. * *p* < 0.05, ** *p* < 0.01, *** *p* < 0.001 compared with the indicated control group.

Based on the aforementioned effect in vitro, C57BL/6 mice were fed a high-fat diet to investigate the therapeutic effect of Rut on diet-induced obesity. After 4 weeks of treatment with 50 mg/kg/day Rut, the body weight gains were markedly lower than the vehicle group (Figure 3A) without altered food intake (Figure S2A). The weights of designated tissues (Figure 3B) and in vivo analysis of body composition (Figure 3C and Figure S2B) showed a lower percentage of fat mass in Rut-treated mice. The adipocyte surface areas of iBAT and iWAT were lower in Rut group (Figure 3D–F), which means less lipid storage. These data show that Rut treatment could reduce lipid storage in adipose tissues and curb diet-induced obesity. 

Metabolic syndrome, characterized by insulin resistance, hyperglycemia and hyperlipidemia, occurs with obesity and critical damage of many tissues. Improvement of insulin resistance and hyperlipidemia often accompany adiposity reduction. HFD feeding induced glucose intolerance and insulin resistance in control mice, while mice subjected to Rut were protected from the abnormal glucose metabolism (Figure 3G–J). Furthermore, the plasma concentrations of total cholesterol (TC), triglyceride (TG) and low-density lipoprotein (LDL), which are indicators of lipid accumulation and metabolic syndrome, were significantly reduced after Rut treatment (Figure S2C–E). All of these data suggest that Rut effectively protects against diet-induced obesity and improves systemic glucose and lipid disorders.

### 2.4. Rut Enhances Energy Expenditure and Adaptive Thermogenesis in HFD-Induced Mice

**Figure 4:**
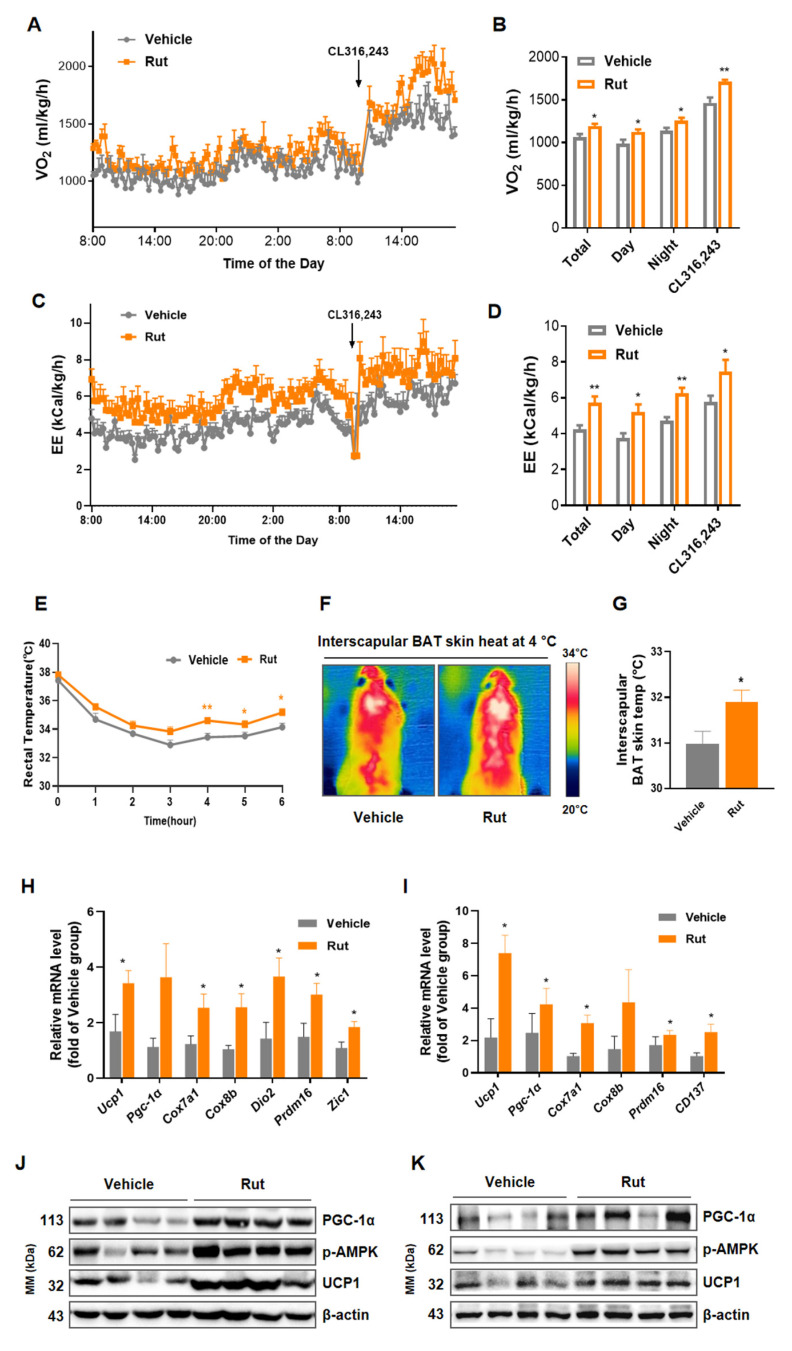
Rut promotes heat generation and energy expenditure in HFD-fed mice. (**A**) Monitoring of oxygen consumption (VO2). (**B**) Average O2 consumption. (**C**) Analysis of energy expenditure (EE) in 24 h and after administration of CL316,243. (**D**) Average energy expenditure. (**E**) Rectal temperatures of HFD mice were recorded every hour after transition to 4 °C from 22 °C. (**F**) Representative infrared thermal images of HFD-fed mice treated with vehicle or Rut after 6 h of exposure to 4 °C. (**G**) Quantification of the interscapular BAT skin temperatures of HFD mice. (**H**,**I**) RT-qPCR analysis of thermogenic genes in iBAT (**H**) and iWAT (**I**) from HFD mice. (**J**,**K**) Western blot analysis of proteins indicated in iBAT (**J**) and iWAT (**K**). *n* = 4–6 per group. Data are presented as the means ±SEM. Student’s *t*-test. * *p* < 0.05, ** *p* < 0.01 compared with the indicated control group.

Balance of food intake and energy expenditure (EE) maintain the whole-body energy homeostasis [32]. Given the unchanged food intake, lower weight of Rut group suggested higher energy expenditure. EE and the oxygen consumption of mice were monitored and analyzed, Rut-treated mice showed higher EE and oxygen consumption ratio under basal and β3-adrenergic receptor agonist CL316,243 stimulation state (Figure 4A–D), but no difference in movement (Figure S2F) was observed between groups. Adaptive thermogenesis plays an important role in body temperature maintenance under cold stimuli. During cold exposure, Rut-treated mice exhibited higher rectal and interscapular BAT skin temperatures (Figure 4E–G). 

Consistent with metabolic phenotype, interscapular brown adipose tissue and inguinal white adipose tissue from Rut-treated mice showed elevated expression levels of thermogenic genes, including *Ucp1*, *Pgc-1α*, *Prdm16*, *Cox7a1*, *Cox8b* and *Dio2*, and browning/beiging specific gene *ZIC1* and *CD137* (Figure 4H,I). Increased UCP1 and PGC-1α protein levels (Figure 4J–K) further verified the effect of Rut on promoting thermogenesis. Taken together, Rut treatment augments whole-body energy expenditure to resist obesity by thermogenic activation of adipose tissue in HFD-fed mice.

### 2.5. Thermoneutrality Blocks the Rut-Induced Thermogenic Program

**Figure 5:**
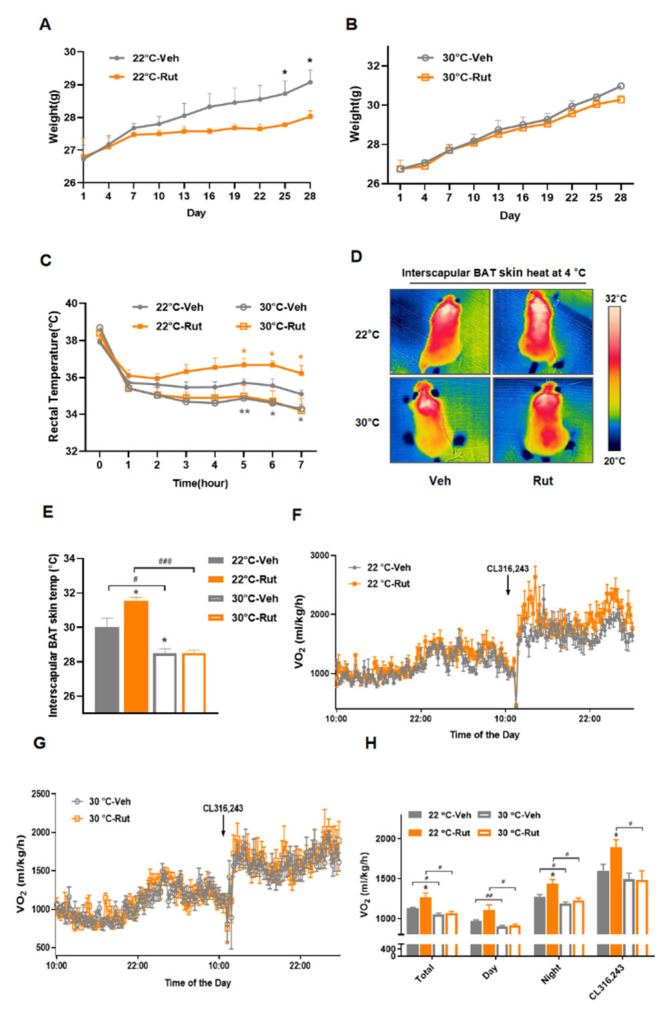
Thermogenic effects induced by Rut treatment are ablated in thermoneutral conditions. (**A**,**B**) Body weights of HFD-fed mice treated with vehicle or Rut housed at 22 °C (**A**) or 30 °C (**B**). (**C**) Rectal temperature changes of mice were recorded every hour during cold stimuli. (**D**) Representative infrared thermal images of mice after cold exposure. (**E**) Quantification of interscapular BAT skin temperatures of 22 °C or 30 °C mice treated with vehicle or Rut. (**F**,**G**) The changes in O_2_ consumption of mice acclimated at 22 °C (**F**) and 30 °C (**G**). (**H**) Assessment of average O_2_ consumption. *n* = 5 per group. Data are presented as the means ±SEM. * *p* < 0.05, ** *p* < 0.01, Rut group versus vehicle group; # *p* < 0.05, ## *p* < 0.01, ### *p* < 0.001, 30 °C group versus 22 °C group by Student’s *t*-test.

Brown and beige adipocytes are viewed “inactive” under thermoneutrality (at 30 °C) that they lose the ability of generating non-shivering thermogenesis to defend cold [33]. To determine the contribution of brown and beige adipocyte thermogenesis in the weight curbing effect of Rut, we housed mice fed a high-fat diet at 30 °C or 22 °C, respectively. At 22 °C, the difference in body weight between Rut-treated and control group was distinct (Figure 5A). However, Rut-treated mice under thermoneutrality gained slightly less body weight than control (Figure 5B). Compared with corresponding group at 22 °C, Rut-treated mice housed at 30 °C were deprived the ability to maintain the core body and interscapular skin temperatures during cold exposure (Figure 5C–E). 

Furthermore, we evaluated the metabolic ratios by measuring the O_2_ consumption. Lower VO2 levels were observed in mice kept at 30 °C, and the elevated O_2_ consumption induced by Rut was blocked under thermoneutrality, either in basal or cold stimuli condition (Figure 5F–H). All these observations indicate that the weight gain-lowering effect of Rut relies on heat production of BAT or thermogenic adipocytes.

### 2.6. The Thermogenesis-Induced Effect of Rut Depends on AMPK/PGC-1α Pathway

**Figure 6:**
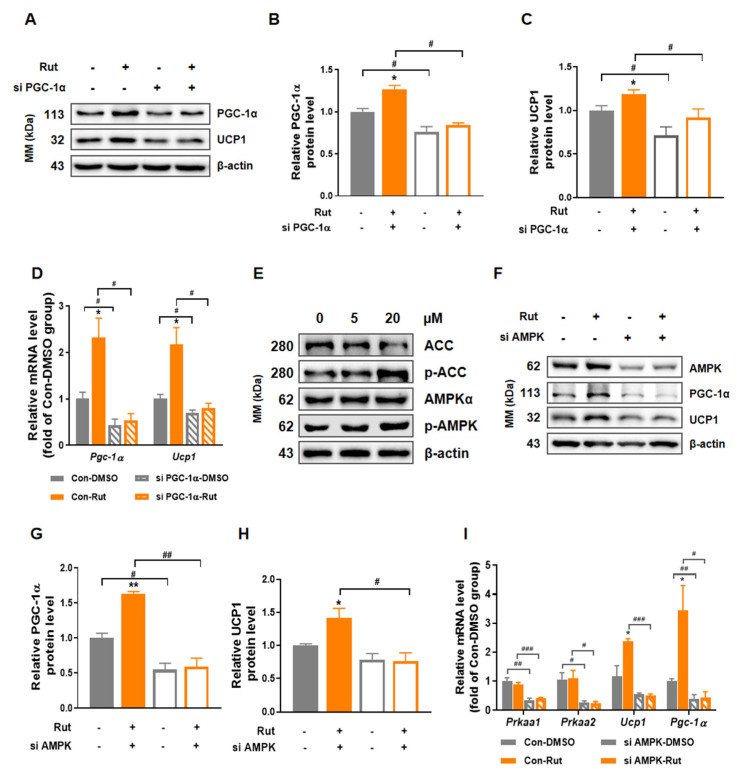
Rut stimulates thermogenic program via AMPK/PGC-1α pathway. (**A**) Expression of PGC-1α and UCP1 protein by siRNA against PGC-1α. (**B**,**C**) Relative protein level of PGC-1α (**B**) and UCP1 (**C**) compared to β-actin. (**D**) The mRNA level of *Pgc-1α* and *Ucp1* after blockage of PGC-1α. (**E**) Expression of p-AMPK, AMPK, p-ACC and ACC protein in C3H10-T1/2 treated by Rut. (**F**) Expression of PGC-1α and UCP1 protein in C3H10-T1/2 with siRNA against AMPK and Rut treatment. (**G**,**H**) Relative protein level of PGC-1α (**G**) and UCP1 (**H**) compared to β-actin. (**I**) The mRNA level of *Pgc-1α* and *Ucp1* after silence of AMPK. *n* = 3 per group. Data are presented as the means ±SEM. * *p* < 0.05, ** *p* < 0.01, Rut group versus vehicle group; # *p* < 0.05, ## *p* < 0.01, ### *p* < 0.001, siRNA group versus control group by Student’s *t*-test.

The aforementioned results imply that Rut ultimately stimulates the adipose thermogenic responses via PGC-1α and downstream targets. To determine the role of PGC-1α in the thermogenic effect of Rut, we blocked PGC-1α in C3H10-T1/2 with small interfering RNA and treated it with Rut. The result showed that blockade of PGC-1α blunted the increased expression of UCP1 induced by Rut in both protein (Figure 6A–C) and mRNA level (Figure 6D), which confirmed that the thermogenic effect of Rut in adipocytes considerably depends on PGC-1α.

To further explore the underlying mechanism, we investigated the canonical thermogenic pathways in adipocytes and found AMPK was activated under Rut treatment (Figure 6E). Then, the up-regulation of UCP1 and PGC-1α protein levels by Rut treatment were blocked in AMPKα1/2-depleted cells (Figure 6F–H), as well as mRNA level (Figure 6I). These results support that Rut exerts its thermogenesis-enhancing function through AMPK/PGC-1α pathway.

### 2.7. Deletion of AMPK Impairs Thermogenic Responses Induced by Rut In Vivo

**Figure 7:**
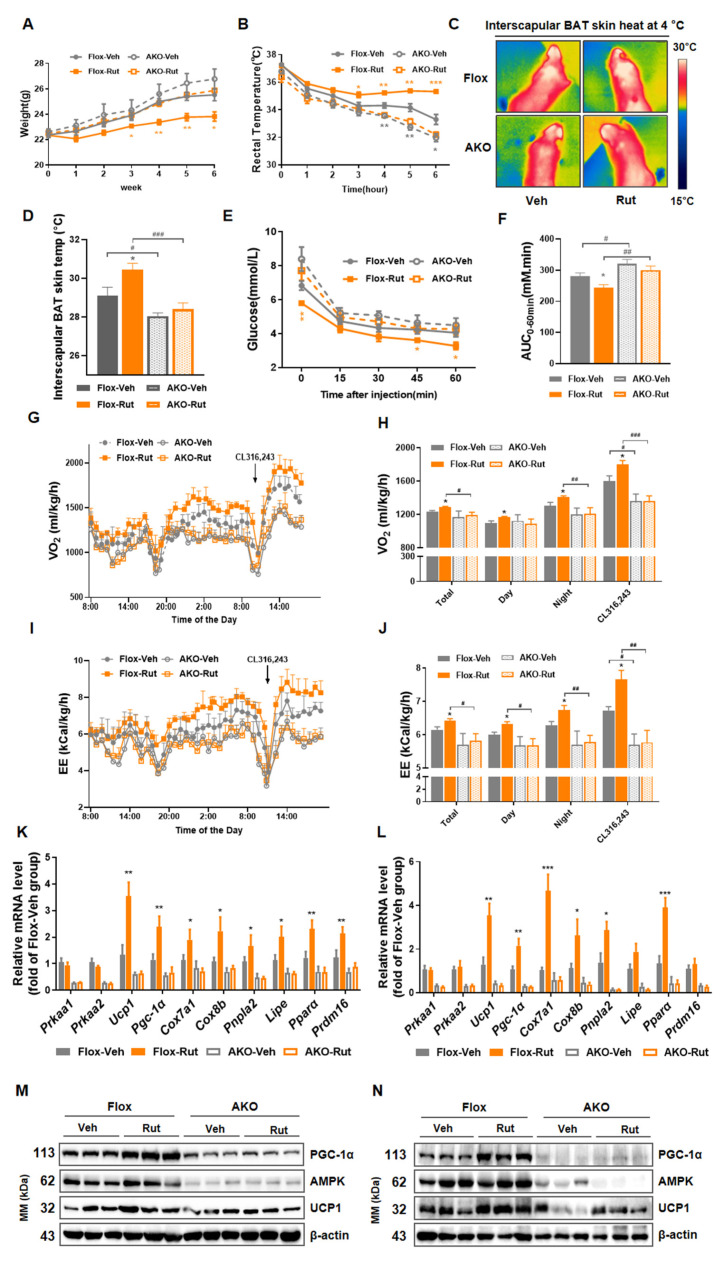
Thermogenic effects induced by Rut are attenuated by absence of AMPK. (**A**) Body weight of the indicated mice group. (**B**) Rectal temperature changes of four groups mice were recorded at 4 °C. (**C**) Representative infrared thermal images of floxed or AKO mice treated with vehicle or Rut after 6 h of exposure to 4 °C. (**D**) Statistical graphs of interscapular BAT skin heat. (**E**) Glucose levels were plotted versus times post insulin injection. (**F**) Analysis of AUC during ITT. (**G**) Testing of O_2_ consumption. (**H**) Average of O_2_ consumption under basal and CL316,243 stimulation conditions. (**I**) Assessment of EE. (**J**) Average of EE under basal and CL316,243 stimulation conditions. (**K**,**L**) Expression level of thermogenic genes by RT-qPCR analysis in iBAT (**K**) and iWAT (**L**). (**M**,**N**) Western blot analysis of AMPK, PGC-1α and UCP1 expression level in iBAT (**M**) and iWAT (**N**) of different groups mice. *n* = 5–6 per group. Data are presented as the means ±SEM. Student’s *t*-test. * *p* < 0.05, ** *p* < 0.01, *** *p* < 0.001, Rut group versus vehicle group; # *p* < 0.05, ## *p* < 0.01, ### *p* < 0.001, AKO group versus floxed group.

Based on AMPK activation of Rut, we generated adipocyte-specific AMPK knockout mice (referred as AKO mice) to investigate whether Rut regulates the adipose tissues thermogenesis and resists obesity via AMPK signaling pathway. Consistent with previous studies [20,34], AKO mice were prone to gain more weight than floxed mice. Moreover, metabolic benefits of Rut treatment were not observed in AKO mice, including less body weight (Figure 7A), increased cold tolerance (Figure 7B–D), improved glucose metabolism (Figure 7E,F and Figure S3A,B), elevated oxygen consumption (Figure 7G,H) and augmented energy expenditure (Figure 7I,J). Under Rut exposure, the mRNA expression of genes involved in thermogenic program were not enhanced in iBAT or iWAT with AMPK deficiency (Figure 7K,L). In parallel, the Rut-induced elevated expressions of UCP1 and PGC-1α in protein level were also blocked in AKO mice, either in iBAT or in iWAT (Figure 7M,N). In this part, the results demonstrate that AMPK deficiency in adipose tissue deprives Rut of the effects on metabolic regulation, which proves the view that AMPK is indispensable for Rut to control weight and benefit metabolism.

In conclusion, all of the data provide systematic evidence that natural product Rut improves thermogenesis in brown and white adipose tissues, promotes energy expenditure and curbs obesity via the adipose AMPK/PGC-1α signaling pathway.

## 3. Discussion

The global epidemic of obesity has increased the burden of social medical care and caused serious harm to human somatic and mental health. Brown and beige adipose depot, due to thermogenesis, represent a promising therapeutic strategy to treat obesity. Three methods could be employed for therapeutic targeting of human thermogenic adipocytes: promoting brown preadipocyte differentiation, governing thermogenesis through regulatory pathways, and increasing WAT browning through the promotion of beige adipocyte formation [35]. Previous studies have demonstrated that numerous molecules possess the potential of combating obesity, including β-adrenergic agonists CL-316,243 [36] and mirabegron [37], cytokines fibroblast growth factor-21 (FGF21) [38], irisin [39] and bioactive ingredients resveratrol [40], berberine [19], etc. In this paper, we found another natural product, Rut, exerts a positive effect on HFD-induced weight gain by regulating thermogenesis in brown and beige adipocytes. Furthermore, our data showed that thermogenesis is indispensable in the anti-obesity role of Rut, as the mass weight of mice between Veh- and Rut-treated groups showed no distinct difference with thermoneutrality. This work adds another salient case for targeting adipose thermogenesis activation as an obesity and related metabolic disease therapy.

It has been well studied that the co-transcriptional factor PGC-1α is closely involved in stimulating the thermogenic program of adipose tissue [41]. In the process of generating heat in brown and beige adipocytes, PGC-1α interacts with other nuclear hormone receptors, such as PPARα, retinoic acid receptor and thyroid receptor, to enhance UCP1 expression. PGC-1α null mice exhibit abnormally increased body fat and glucose homeostasis and these mice were unable to maintain core body temperature following exposure to cold [42]. Conversely, over-expression of PGC-1α is sufficient to increase mitochondrial number and function [43]. In mammals, PGC-1α is physiologically induced by conditions of shortage of, or increased demand for, energy, such as cold, physical activity and fasting [44]. Besides physiological activation, PGC-1α can also be stimulated pharmacologically. Previous studies suggest numerous phytochemicals play a beneficial thermogenesis role via PGC-1α induction, such as berberine [19], Curcumin [45], resveratrol [46], epigallocatechin gallate (EGCG) [47], EPA [48,49] and all-trans retinoic acid (ATRA) [50,51]. Consistent with previous views, Rut, an activator of PGC-1α, showed to possess great ability to trigger thermogenesis here. Moreover, knockdown of PGC-1α in vitro indicated that PGC-1α is positively involved in the enhancement of thermogenesis induced by Rut in brown adipocytes. 

In our further mechanistic studies, energy sensing kinase AMPK made a very positive contribution to the effect of Rut in vitro and in vivo. As an energy regulator, a functional upregulation of AMPK in adipocytes benefits from the thermogenic responses of brown and beige fat and improves diet-induced glucose intolerance [52]. In agreement with our previous work [34], we found that adipose tissue-specific AMPK deficiency mice gain more weight and suffer from insulin resistance. For Rut, we showed direct evidence that AMPK is indispensable for its positive effect on whole body glucose and energy homeostasis. Recently, this finding was also reported by Surbala et al., i.e., that Rut increased the activity of AMPK in hepG2 cells and decreased the elevated blood glucose in HFD-fed multiple-dose streptozotocin-induced type 2 diabetes mellitus mice. Even though there was no clear answer as to whether Rut activates AMPK directly here, some clues could be found from previous research. In endothelial cells, AMPK is activated by the upstream kinases CaMKII and CaMKKβ in the action of Rut-induced endothelial nitric oxide synthase (eNOS) activation and nitric oxide (NO) generation. Thus, it is reasonable to assume that Rut is more likely to regulate AMPK activity in an indirect way.

The AMPK/PGC-1α pathway has been reported many times before in the process of heat generation and browning, which indicates that this signaling pathway plays a crucial role in initiating thermogenic program and controlling energy metabolism. Recently, an interesting study reported that N-butylidenephthalide upregulates the expression of thermogenic markers, ameliorates hyperlipidemia/hyperglycemia, and increases metabolic activity through fat browning, and these are regulated by AMPK/PGC-1α [53]. Additionally, natural molecule berberine has been demonstrated to regulate a thermogenesis program in both BAT and WAT, and these effects are AMPK/PGC-1α dependent [19]. In this paper, we highlight once again the significance of AMPK/PGC-1α pathway in the heat-raising process through the action of Rut. The in vitro and vivo data suggested that destruction of the pathway leads to much or all loss of RUT-induced fat combustion.

Rut has long been used for the treatment of gastrointestinal disorders, headache, amenorrhea and postpartum hemorrhage in traditional oriental medicine [25]. It has received sustained attention because of its numerous interesting biological properties. Yet, the functions of Rut in metabolic diseases are less systematic investigated. Herein, the excellent properties of Rut in promoting energy consumption and defeating obesity had been identified. Mechanistically, results suggested activation of AMPK/PGC-1α pathway is responsible for the Rut-induced salutary metabolic phenotypes. A recent study has reported that Rut promotes beiging of white adipocyte through activation of the AMPK-PRDM16 axis [54]. Our findings indicated primary adipocytes and C3H10-T1/2 cells can be stimulated by Rut to elevate the expression of *Pgc-1α*, *Ucp1* and other thermogenic genes. Along with these, higher oxygen consumption rates were observed in both differentiated brown and beige adipocytes. Our data, however, do not exclude the possibility that the browning of preadipocyte in WAT play a role in Rut-induced metabolic benefits in vivo. Further investigation will help to understand the underlying mechanism of Rut action and promote its application in metabolic regulation.

## 4. Materials and Methods

### 4.1. Materials

The antibody sources were as follows: UCP1 (Abclonal, A5857), PGC1α (Calbiochem, ST1202); AMPKα (Cell Signaling Technology, #2532); phospho-AMPKα (Thr172) (Cell Signaling Technology, #2535); ACC (Cell Signaling Technology, #3662); phospho-ACC (Ser79) (Cell Signaling Technology, #3661); and β-actin (Abgent, AM1021B). 

DMEM/F12 Ham 1:1, high glucose DMEM medium and fetal bovine serum (FBS) were purchased from Gibco (Waltham, MA, USA). Rosiglitazone, 3-Isobutyl-1-methylxanthine (IBMX), 3,3′,5-Triiodo-L-thyronine (T3), indomycine, dexamethasone, oligomycin, carbonyl cyanide 4-(trifluoromethoxy) phenylhydrazone (FCCP), rotenone, antimycin A, cremophor EL collagenase D (Roche) and Dispase II (Roche) were purchased from Sigma Aldrich. Recombinant human Insulin (Novolin) was purchased from Changzheng Hospital in Shanghai, China. Lipofectamine™ 3000 Transfection Reagent and penicillin/streptomycin were from Introvigen (Grand Island, NY, USA). PrimeScript Reverse Transcriptase and PrimeScript Reverse Transcriptase were from Takara (Takara Ltd., Otsu, Japan). SYBR Green qPCR Master Mix wase from ABclonal Technology Co., Ltd (Shanghai, China). RIPA lysis buffer, loading buffer and hematoxylin and eosin (H&E) were purchased from Beyotime Biotechnology Co., Ltd (Shanghai, China).

Kits used in measurement of plasma parameters are as follows: total triglyceride (TG), total cholesterol (TC) and low-density lipoprotein (LDL) were from Shanghai Fosun Long March.

### 4.2. Chemical

Rut (purity 98%) was purchased from Shanghai Macklin Biochemical Co., Ltd (Shanghai, China). (R817266). For the in vitro study, Rut was dissolved in DMSO at the indicated concentration. For the in vivo study, Rut was made up in 1% DMSO, 5% castor oil and 0.5% carboxymethycellulose sodium (CMC-Na) for animal orally administration at 5 mg/mL. All solutions were freshly prepared on the experimental day.

### 4.3. Culture and Drug Treatment

C3H10-T1/2 cells were a gift kindly given by Jiqiu Wang, Ruijin Hospital, and their cells were obtained from ATCC. This is a cloned murine embryo fibroblast cell line, which was isolated from a line of C3H mouse embryo cells. It has the potential of multi-directional differentiation so that it could possess the characteristics of brown adipocytes after specified induction, which is a reliable tool for the study of brown adipocytes. C3H10-T1/2 cells with a passage number less than 20 were used for experiments. Hek293 cells and C3H10-T1/2 cells were cultured in high glucose DMEM supplemented with 10% FBS and 1% PS with medium replacement every two days. When C3H10-T1/2 density reached 80%, the cells were seeded into cell plates. After two days of confluence, C3H10-T1/2 was induced into brown adipocytes with induction medium (basal medium added with 850 nM insulin, 0.5 mM IBMX, 1 μM dexamethasone, 125 nM indomethacin, 1 nM T3 and 1 μM Rosiglitazone) for 2 days and maintenance medium (basal medium added with 850 nM insulin 1 nM T3 and 1 μM Rosiglitazone) for 6 days. Cells were treated with Rut at Day 8 for 24 h.

### 4.4. Cell Transfection

C3H10-T1/2 of Day 6 was transfected with 50 nM of siRNAs targeting PGC1α or AMPKα using Lipofectamine™ 3000 Transfection Reagent (Invitrogen) according to standard methods. After 24 h, the transfection mix was replaced with basal medium, and then compounds were added and incubated for another 24 h. The sequences of siRNA for PGC1α (si PGC-1α) and AMPKα (si AMPKα) were as follows: si PGC-1α-1: GUAGCGACCAAUCGGAAAUTT, AUUUCCGAUUGGUCGCUACTT; si PGC-1α-2: CCGCAAUUCUCCCUUGUAUTT, AUACAAGGGAGAAUUGCGGTT; si PGC-1α-3: CCCACAGGAUCAGAACAAATT, UUUGUUCUGAUCCUGUGG GTT; si AMPKα1: UUUGAAAGACCAAAGUCGGCU, CCGACUUUGGUCUUUCAAACA; si AMPKα2: AUCAAAC UGCGAAUCUUCUG, GAAGAUUCGCAGUUUAGAUGU.

Scrambled siRNA sequences were used as the negative control.

### 4.5. Isolation of Primary Pre-Adipocytes

Stromal vascular fraction (SVF) cells from adipose tissue were isolated as previously described [55]. Cells were isolated from dissected interscapular brown fat or inguinal white fat pad of four to six-week-old mice. After being collected, these fractions were digested with collagenase D and Dispase II. in PBS at 37 °C for 45 min, with oscillation every 15 min. Primary SVF cells were cultured in DMEM/F12 ham 1:1 with 10% FBS and 1% penicillin streptomycin until confluence. The differentiation induction was similar to C3H10, except for 1 μM T3 in its induction and maintenance medium.

### 4.6. Quantitative RT-PCR Analysis (RT-qPCR)

Total RNA from indicated cells and tissues was lysed using Trizol regent according to manufacturer’s protocols. The purity and concentration of RNA extracted were assessed by Nanodrop 2000 (Thermo Fisher Scientific, Waltham, MA, USA). Moreover, 500 μg of RNA was reverse transcribed using PrimeScript Reverse Transcriptase. Transcription reactions were incubated for 15 min at 37 °C water bath and then inactivated by incubation at 85 °C for 5 s. Afterward, the synthesized cDNA mixtures were further diluted and reacted using SYBR Green qPCR Master Mix a Stratagene Mx3005P instrument (Agilent Technologies, Santa Clara, CA, USA). The PCR program was: 95 °C for 5 min, 40 cycles of 95 °C 30 s/60 °C 30 s/72 °C 30 s, and finally 72 °C for 5 min. Relative mRNA levels were calculated using the ΔΔCT method and normalized to the housekeeping gene 36b4. The primer sequences are listed in Supplementary Table S1. 

### 4.7. Western Blotting

Total proteins from cells or tissues were lysed by RIPA lysis buffer and denatured with loading buffer. Equal amounts (20 μg) of protein per samples were electrophoresed in 10% SDS-PAGE at 80 volts for 30 min and 120 volts for 60 min to separate protein bands. The proteins were transferred to a nitrocellulose filter membrane using a wet transfer setup under 100 volts for 90 min. Membranes were blocked in TBS-T (10 mM Tris-HCl pH 7.4, 150 mM NaCl, 0.05% Tween-20), containing 5% (*w/v*) non-fat dried skimmed milk powder for 1 h and then incubated with primary overnight at 4 °C. Primary antibodies were used at a concentration of 1 µg/mL except β-actin antibody at 0.2 µg/mL. After washing with TBS-T for three times, blots were incubated with secondary antibodies for 1 h at room temperature. The blots were detected with enhanced chemifluorescent substrate (Epizyme Biomedical Technology Co., Shanghai, China). The relative expression of protein was quantified by the densitometric analysis with Image J software (National Institutes of Health).

### 4.8. Measurement of Mitochondrial Respiratory Capacity

Mitochondrial respiratory function was detected by measuring the oxygen consumption rate (OCR). Differentiated primary adipocytes were plated in an XF 96-well microplate (Seahorse Bioscience) in the manufacturers’ designated culture/assay plate and then treated with corresponding concentration Rut for 24 h. Mitochondrial respiratory capacity was detected by measuring the oxygen consumption rate (OCR). Additionally, 2 μM oligomycin, 1 μM FCCP and 1 μM rotenone/antimycin A were used to detect uncoupled respiration, maximal respiration and nonmitochondrial respiration, respectively.

### 4.9. Animals

All animal procedures followed were approved by the institutional animal care and use committee. C57BL/6 male mice (Beijing HFK Bioscience Co., Ltd., Beijing, Shanghai, China) were housed at 22 ± 2 °C under a 12–12 h light/dark cycle with free access to food and water until 10 weeks of age. After 6 weeks of exposure to a high-fat diet (60% kcal from fat, D12492, Research Diets), the mice were randomly divided into two groups of control (1% DMSO and 5% castor oil in 0.5% CMC-Na) and Rut (50 mg/kg), with six mice per group. Drugs were given by gavage administration to their corresponding groups once a day continuously for 7 weeks. Six additional age-matched mice of NC group were fed normal chow. For the thermoneutral experiment, 8-week male mice were randomized and kept at 22 °C or 30 °C, respectively. After a week accumulation, drugs were administrated to corresponding group. During the experiment, mice body weights were recorded every day. Adipose tissue-specific AMPKα1/α2-KO mice (AKO) were generated as previously described [34]. At 8 weeks of age, AKO mice and age-matched AMPKα1/α2-floxed littermates were with vehicle or Rut. At 14 weeks of age, tissues were collected, weighed and stored at −80 °C.

### 4.10. Histology

Adipose tissues were fixed in 4% formaldehyde overnight at room temperature, embedded in paraffin, and sectioned (6 μm) by a microtome. Blocks were stained using hematoxylin and eosin (H&E) according to the manufacturer’s instructions. The pictures of sections obtained by a Leica DM6 B microscope and cell surfaces were calculated by ImageJ software.

### 4.11. Metabolic Study

Body composition (fat mass, lean mass and fluid) was assessed by using a magnetic resonance whole-body composition analyzer (Minispec LF90 II, Bruker, Karlsruhe, Germany). Oxygen consumption, carbon dioxide production and locomotor activity were measured using a sixteen-chamber indirect calorimeter (TSE PhenoMaster) according to the manufacturer’s instructions [56]. The mice were maintained at 22 °C under a 12 h light-dark cycle following a 12 h acclimatization. To obtain metabolic parameters under cold stimuli, mice were intraperitoneally injected with the β3-adrenergic receptor-specific agonist CL316,243 (1 mg/kg) after a 12 h light/dark cycle. The mice were given free access to water and food. Heat production as name as energy expenditure, oxygen expenditure and activity were calculated as described previously. For cold exposure, mice were single-caged and exposed to a temperature of 4 °C for 6 h. Rectal temperature was monitored every hour using a BAT-12 microprobe digital thermometer and RET-3 mouse rectal probe (Physitemp Instruments, Clifton, NJ, USA). Thermo images were taken using an E6 Thermal Imaging Infrared Camera (E6, FLIR Systems, Portland, OR, USA) at 6 h, and the interscapular skin temperature was analyzed using FLIR Tools software.

### 4.12. Glucose Tolerance Test (GTT) and Insulin Tolerance Test (ITT)

For C57BL/6 high-fat diet-induced mice, GTT and ITT were performed 5 and 6 weeks after drug administration, respectively. Mice were weighted and blood glucose was measured as a basal glucose value after fasting 6 h for GTT and 4 h for ITT. Then, mice were provided with glucose (2.5 g/kg, p.o.) or insulin (0.75 U/kg, i.p.) and blood glucose was recorded at various time points after injection (15, 30, 60, 90 and 120 min). The area under curve (AUC) for glucose was calculated and compared between the groups. For AMPKα1/α2-KO mice, GTT and ITT were performed 5 and 6 weeks, respectively. Mice were provided with glucose (2.5 g/kg, p.o.) or insulin (0.5 U/kg, i.p.) and blood glucose were recorded at various time points after injection (15, 30, 60, 90 and 120 min) for GTT and (15, 30, 45 and 60 min) for ITT.

### 4.13. Statistical Analysis

Data are expressed as the mean ±SEM. Normality was tested with the Shapiro–Wilk normality test. Two-tail Student’s *t*-test was used for comparisons between two groups and one-way ANOVA was used for comparisons of more than two groups followed by Tukey’s multiple comparisons test. Significance was considered as *p* < 0.05. Analyses and figures were produced using GraphPad Prism software (version 7.00, GraphPad Software, La Jolla, CA, USA).

## 5. Conclusions 

**Figure 8:**
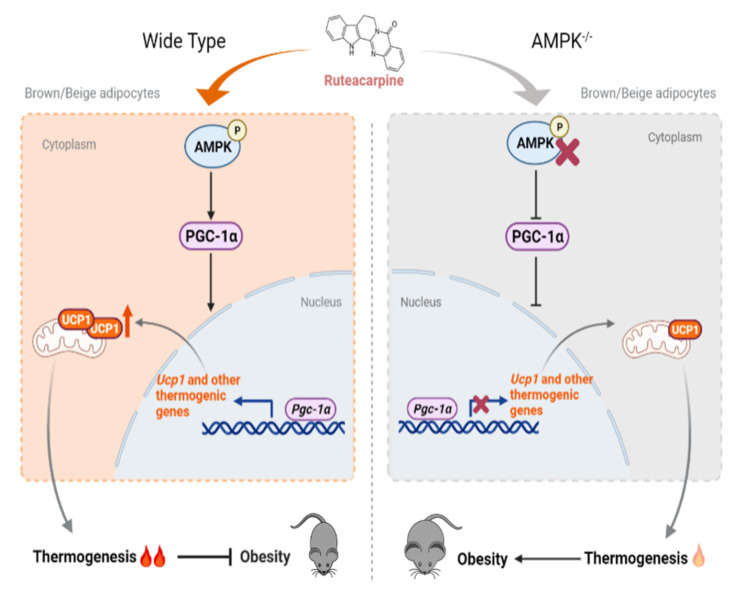
Graphical abstract. In this study, we revealed the effects of rutaecarpine (Rut) on thermogeneis and weight management. Rut can reduce adiposity, improve insulin resistance and increase respiratory activity in mice with HFD-induced obesity. The mechanism of the effects of Rut involved AMPK/PGC-1α pathway, and we demonstrated that AMPK deficiency in adipose tissue suppressed the upregulation of thermogenic markers and beneficial metabolic effects in Rut-treated mice.

Combined with our work (Figure 8), Rut could regulate thermogenesis program in both brown and beige adipocytes, and these effects are AMPK/PGC-1a dependent. The in vivo study strongly demonstrates a protective role of Rut in restraining obesity and related metabolic morbidities without affecting food intake or lean mass, as well as the positive impacts of Rut on weight control and energy expenditure due to the thermogenic process of adipose tissues. Collectively, these findings support the further development of Rut as a potential therapeutic for obesity and metabolic diseases.

## Data Availability

Data is contained within the article and supplementary material.

## Supplementary Materials

The following supporting information can be downloaded at: https://www.mdpi.com/article/10.3390/ph15040469/s1, Table S1: Primer sequences used in real time RT-qPCR (mouse); Figure S1: Rut has a satisfactory safety and increases mitochondrial copy number of adipocytes; Figure S2: Rut improves lipid accumulation and enhances energy expenditure without altered food intake and locomotor activity; Figure S3: AMPK deficiency fails to achieve Rut-induced glucose tolerance improvement.

## Abbreviations

PGC1α: Peroxisome proliferator-activated receptor gamma coactivator 1-alpha; UCP1: uncoupling protein 1; AMPK: AMP-activated protein kinase; BAT: brown adipose tissue; WAT: white adipose tissue; Cidea: cell death-inducing DFFA-like effector a; Cox7a1: cytochrome c oxidase polypeptide 7a1; Cox8b: cytochrome c oxidase polypeptide 8b; CPT1a: carnitine palmitoyltransferase 1a; Dio2: type II iodothyronine deiodinase; Acox1: acyl-CoA oxidase 1; Pparα: peroxisome proliferator activated receptor alpha; Mcad: medium-chain acyl-coenzyme A dehydrogenase; PRDM16: PR domain containing 16; ACC: acetyl-CoA carboxylase; iWAT: inguinal white adipose tissue; eWAT: epididymal white adipose tissue; GTT: glucose tolerance test; ITT: insulin tolerance test; H&E: hematoxylin and eosin; HFD: High-fat diet; SVF: stromal vascular fraction; T3: 3,3′,5-Triiodo-L-thyronine; IBMX: 3-Isobutyl-1-methylxanthine; OCR: oxygen consumption rates; EE: energy expenditure; RER: respiratory exchange rate; TC: total cholesterol; TG: triglyceride; LDL: low-density lipoprotein.
