# Supplementary material for: Rutaecarpine Promotes Adipose Thermogenesis and Protects against HFD-Induced Obesity via AMPK/PGC-1α Pathway
Source: Pharmaceuticals (Basel). 2022 Apr 13;15(4):469. doi: 10.3390/ph15040469 (PMC9027001; doi:10.3390/ph15040469)
Supplement: Supplementary file 1 [file pharmaceuticals-15-00469-s001.zip › pharmaceuticals-1637500-supplementary.pdf]

Supplementary Table S1. Primer sequences used in real time Q-PCR (mouse).

Genes	Sequence
36b4 forward	TTTGGGCATCACCACGAAAA
36b4 reverse	GGACACCCTCCAGAAAGCGA
Prkaa1 forward	AAAGTGAAGGTGGGCAAGCA
Prkaa1 reverse	CAGATGGTGTACTGATGACCTGG
Prkaa2 forward	TCGCAGTTTAGATGTTGTTGGA
Prkaa2 reverse	CTTCAACCCGCCCATGTTTG
Ucp1 forward	ACTGCCACACCTCCAGTCATT
Ucp1 reverse	CTTTGCCTCACTCAGGATTGG
Pgc-1α forward	ACTGAGCTACCCTTGGGATG
Pgc-1α reverse	TAAGAATTTCGGTGGTGACA
Prdm16 forward	CAGCACGGTGAAGCCATTC
Prdm16 reverse	GCGTGCATCCGCTTGTG
Cox7a1 forward	CAGCGTCATGGTCAGTCTGT
Cox7a1 reverse	AGAAAACCGTGTGGCAGAGA
Pnpla2 forward	GGTGACCATCTGCCTTCCAG
Pnpla2 reverse	TGCAGAAGAGACCCAGCAGT
Mcad forward	AGCTGCTAGTGGAGCACCAAG
Mcad reverse	TCGCCATTTCTGCGAGC
Elovl3 forward	TTCTCACGCGGGTTAAAAATGG
Elovl3 reverse	GAGCAACAGATAGACGACCAC
Cox8b forward	GAACCATGAAGCCAACGACT
Cox8b reverse	GCGAAGTTCACAGTGGTTCC
Dio2 forward	AATTATGCCTCGGAGAAGACCG
Dio2 reverse	GGCAGTTGCCTAGTGAAAGGT
Cidea forward	TGCTCTTCTGTATCGCCCAGT
Cidea reverse	GCCGTGTTAAGGAATCTGCTG
Pparα forward	AGGCCGTTGCCACTGTTCAG
Pparα reverse	AGCCCTCTTCATCCCCAAGC
18S rRNA forward	AGT CCC TGC CCT TTG TAC ACA
18S rRNA reverse	CGATCCGAGGGCCTCACTA
16S rRNA forward	CCGCAAGGGAAAGATGAAAGAC
16S rRNA reverse	TCGTTTGGTTTCGGGGTTTC
ATP6 forward	CTATTCCCATCCTCAAAACG
ATP6 reverse	CTTTTGGTGTGTGGATTAGC
COX1 forward	GCTAGCCGCAGGCATTACTA
COX1 reverse	CTCCTCCAGCGGGATCAAAG
ND3 forward	GTTGCATTCTGACTCCCCCA
ND3 reverse	GGTAGACGTGCAGAGCTTGT

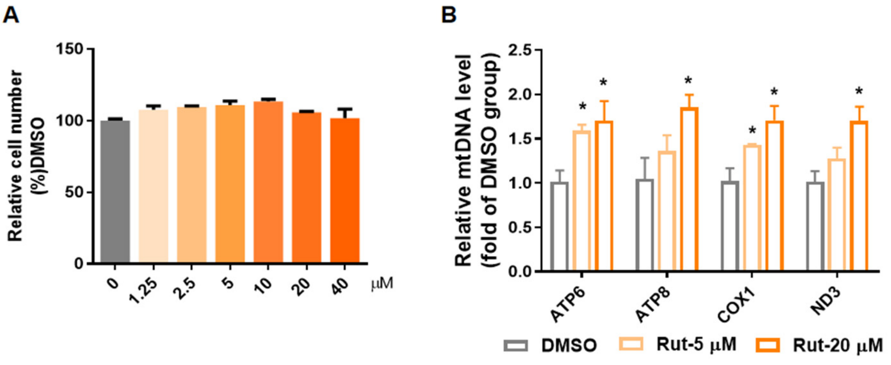

Figure S1. Rut has a satisfactory safety and increases mitochondrial copy number of adipocytes. (A) Cell vitality after different concentration Rut treatment. (B) Mitochondrial copy number of C3H10-T1/2 with Rut treatment.The copy number of mtDNA was normalized to nuclear DNA (18S ribosomal RNA). n=3 per group. Data are presented as the means ± SEM. *P < 0.05, Rut groups versus vehicle group by one-way ANOVA.

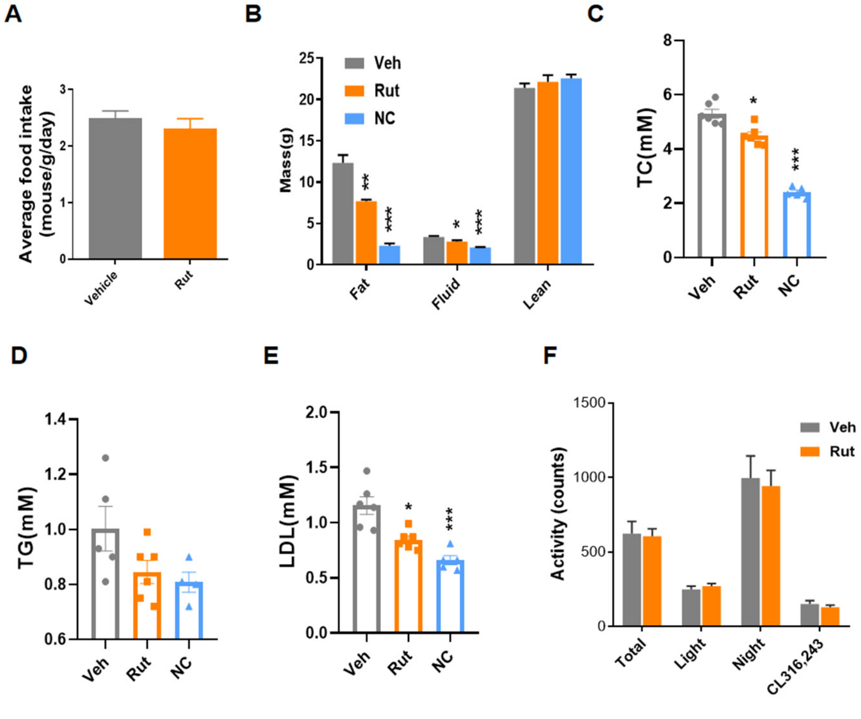

Figure S2. Rut improves lipid accumulation and enhances energy expenditure without altered food intake and locomotor activity. (A) Average food intake of HFD mice. (B) Body composition of HFD mice. (C-E) plasma concentrations of total cholesterol (TC), triglyceride (TG) and low-density lipoprotein (LDL). (F) Monitoring of mice activity in basal and cold stimuli condition. n=3-6 per group. Data are presented as the means ± SEM. Student's t-test. *P < 0.05, **P < 0.01, ***P < 0.001 compared with the indicated control group.

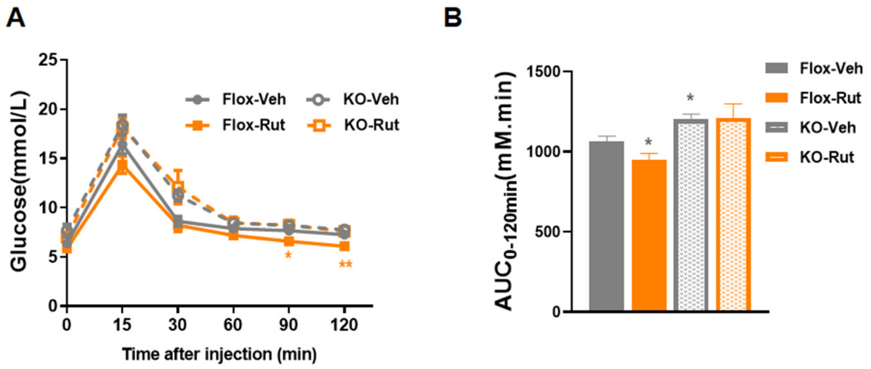

Figure S3. AMPK deficiency fails to achieve Rut-induced glucose tolerance improvement. (A) Blood glucose during OGTT. (B) Area under OGTT curve. n=5-6 per group. Data are presented as the means ± SEM. Student's t-test. *P < 0.05, **P < 0.01 compared with the indicated control group.
